# Urea/Creatinine Ratio’s Correlation with Creatine Kinase Normalization in Pediatric COVID-19 Patients with Myositis: Evaluating Prognostic and Predictive Value

**DOI:** 10.3390/idr16010002

**Published:** 2023-12-25

**Authors:** Francesco Pizzo, Andrea Marino, Alessandra Di Nora, Serena Spampinato, Giovanni Cacciaguerra, Giuseppe Costanza, Federica Scarlata, Arturo Biasco, Maria Chiara Consentino, Riccardo Lubrano, Bruno Cacopardo, Giuseppe Nunnari, Martino Ruggieri, Piero Pavone

**Affiliations:** 1 Postgraduate Training Programme in Pediatrics, Department of Clinical and Experimental Medicine, University of Catania, Via S. Sofia 78, 95123 Catania, Italy; pifrance@hotmail.it (F.P.); alessandradinora@gmail.com (A.D.N.); gio.cacciaguerra@gmail.com (G.C.); giuseppe93costa@gmail.com (G.C.); scarlatafederica8@gmail.com (F.S.); arturobiasco94@gmail.com (A.B.); mchiara.cosentino@gmail.com (M.C.C.); 2 Infectious Diseases Unit, Department of Clinical and Experimental Medicine, ARNAS Garibaldi Hospital, University of Catania, 95122 Catania, Italy; cacopard@unict.it (B.C.); giuseppe.nunnari1@unict.it (G.N.); 3 Department of Clinical and Experimental Medicine, University of Messina, 98122 Messina, Italy; serenaspampinato93@gmail.com; 4 Department of Paediatrics, Sapienza University of Rome, Viale del Policlinico 155, 00161 Roma, Italy; riccardo.lubrano@uniroma1.it; 5 Department of Clinical and Experimental Medicine, Section of Pediatrics and Child Neuropsychiatry, University Hospital “Policlinico G. Rodolico”, 95125 Catania, Italy; m.ruggieri@unict.it

**Keywords:** SARS-CoV-2 infection, pediatric COVID-19, COVID-19 myositis, COVID-19 myolysis

## Abstract

Coronavirus disease 2019 (COVID-19) has been chiefly linked with substantial respiratory complications. However, emerging studies have brought attention to the occurrence of severe muscle inflammation (myositis) related to COVID-19, potentially leading to multi-organ failure and increased mortality. Myositis is generally characterized by heightened serum creatine kinase (CK) levels. Acute myositis is characterized by an infiltration of viruses into calf muscle fibers, which may cause a subsequent inflammatory response leading to calf muscle pain. Symptomatic and supportive management, along with explanation and reassurance, is all that is required in managing this condition. While the association between myositis and severe outcomes has been recognized in adults, it remains less understood in the pediatric population. The current retrospective study, conducted at Policlinico San Marco University Hospital in Catania, aimed to analyze clinical and laboratory factors associated with myositis in pediatric patients with SARS-CoV-2 infection. Between January 2022 and January 2023, ten pediatric patients diagnosed with myositis and SARS-CoV-2 infection were evaluated. The study highlighted clinical manifestations such as fever, calf muscle pain, and abnormal gait. Lab results showed elevated CK levels among other findings. All patients underwent treatment, with the majority recovering without complications. A notable correlation was observed between CK levels, blood urea nitrogen (BUN), and the urea/creatinine ratio (UCR). The study also discusses potential pathophysiological mechanisms behind SARS-CoV-2’s impact on skeletal muscles, emphasizing an indirect inflammatory response. Our findings underscore that while myositis in children with SARS-CoV-2 infection appears to follow a benign and self-limiting trajectory, it is crucial to monitor specific markers for early intervention and management. Further research is warranted to elucidate the underlying mechanisms and improve clinical outcomes.

## 1. Introduction

The emergence of the COVID-19 pandemic has posed an unprecedented challenge to the global healthcare community. Originating in Wuhan, China, in December 2019, the disease has shown its primary effects through respiratory complications, as caused by the severe acute respiratory syndrome coronavirus 2 (SARS-CoV-2) [1]. The number of confirmed cases has surged dramatically, leading to substantial morbidity and mortality [2,3,4].

Originally, the primary symptoms of COVID-19 were identified as fever, cough, and difficulty breathing. These respiratory symptoms were the first indication of its detrimental effects [1]. However, with time and extensive research, it became evident that the disease had far-reaching implications beyond the respiratory system. This was illuminated through a myriad of uncommon clinical manifestations that have come to the fore, as detailed in a range of studies [5,6]. One of these manifestations is severe muscle inflammation and injury related to the disease. These musculoskeletal complications, although initially overshadowed by the more predominant respiratory symptoms, began to gain increased attention due to their potential to trigger multi-organ failure and even death [7,8,9,10,11,12,13,14].

It is essential to differentiate between myositis and myolysis when discussing muscle-related complications. Myositis refers to the inflammation of muscles, which can result from various causes, including viral infections, autoimmune reactions, or medications, childhood-onset or juvenile idiopathic inflammatory myopathies (JIIMs), and metabolic myopathies. On the other hand, myolysis is characterized by an infiltration of viruses into calf muscle fibers, which may cause a subsequent inflammatory response leading to calf muscle pain. This degradation often leads to the release of a protein called myoglobin into the bloodstream, which can subsequently result in kidney damage if not properly addressed. While both conditions involve muscle complications, their underlying causes, manifestations, and potential consequences are distinct [15].

Regarding the influence of SARS-CoV-2 on muscle tissue, researchers have hypothesized two primary pathways: a direct interaction via the ACE2 receptor and an indirect one involving an intense inflammatory response within the muscle [16]. Research by dos Santos et al. has indicated that human skeletal muscle does not express the ACE-2 gene, as per single-cell RNA sequence analysis. Their findings also point out that muscle breakdown occurred after the reduction of fever, hinting that the indirect pathway may be primarily responsible for muscle impairment associated with SARS-CoV-2 [16].

Elevated levels of certain cytokines during the infection, such as IL-6, IL-1β, IL-8, IFN-γ, IP-10, and TNF-α, are believed to cause muscle degradation and hinder protein synthesis [16,17,18]. Further, a histological examination of the psoas muscles in deceased patients revealed MHC-1-positive staining in muscle fibers and infiltration by CD68-, CD4-, and CD8-positive histiocytes and T cells [19]. These findings highlight that muscle damage in SARS-CoV-2 cases may be a consequence of inflammatory reactions, particularly due to cytokine-induced injury. 

Myositis, a term that perhaps was not commonly associated with viral infections, suddenly became a topic of interest for many clinicians and researchers. Characterized by a serum creatine kinase (CK) level exceeding certain limits, it served as an indicator of muscle injury [15].

Pediatric COVID-19 patients showed different patterns, with some adolescents presenting with severe renal impairments but a lower association with myolysis [20,21,22].

To understand why and how these complications occur, we need to dive deep into the pathophysiology of the virus [23]. The virus’s mode of action involves targeting cells that have ACE-2 receptors [16]. These receptors are present in many organs, including the lungs, heart, kidneys, and muscles. The binding of the virus’s spike protein to these receptors, especially in muscle cells, can cause a series of reactions leading to inflammation. This inflammation can result in tissue damage and, in severe cases, myolysis.

Furthermore, the immune response triggered by the virus can sometimes go into overdrive, leading to what is known as a ‘cytokine storm’. This intense immune reaction can aggravate the muscle damage, further complicating the clinical scenario [16].

Considering these findings, early diagnosis and management of these musculoskeletal symptoms become paramount. The need to identify clinical and laboratory prognostic factors is more critical than ever. Such knowledge can guide clinicians in managing the disease more effectively, ensuring better patient outcomes [16].

As the world continues to grapple with COVID-19, understanding its full spectrum is of utmost importance. This study, focusing on musculoskeletal pediatric complications in children with SARS-CoV-2 infection, is a step in that direction.

## 2. Materials and Methods

This retrospective study was conducted at Policlinico San Marco University Hospital in Catania. We included only patients under 18 years old who tested positive for SARS-CoV-2 using a nasal swab and were subsequently hospitalized. A retrospective analysis was performed on patients with acute myositis between January 2022 and January 2023. 

### 2.1. Patient Enrollment

**Figure 1:**
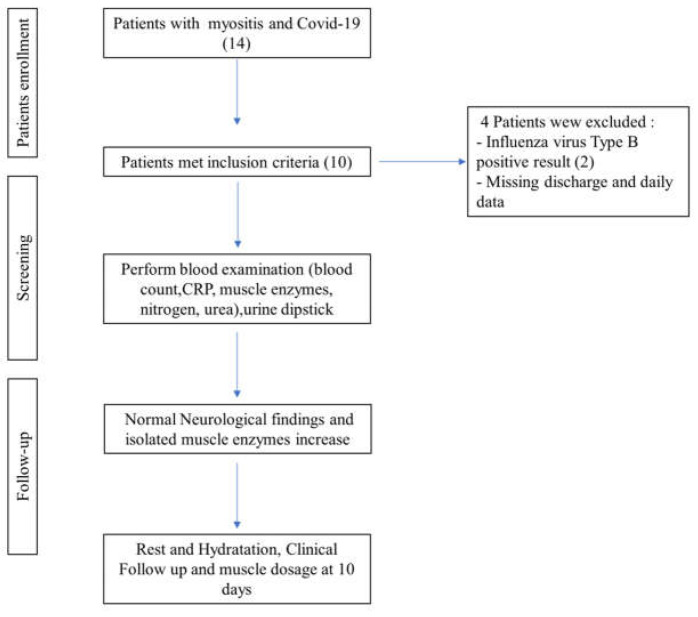
Patients’ clinical enrollment.

We enrolled patients aged 1 to 14 years who presented with clinical symptoms suggestive of myositis, an acute (48 h) elevation of creatine kinase (CK) levels, approximately 5 to 10 times the normal limit, and a positive SARS-CoV-2 swab result (Figure 1).

### 2.2. Clinical Management Pathway

Each patient underwent clinical examinations and was screened for respiratory viruses using nasal swabs (BIOFIRE, FILMARRAY Respiratory Panel, BioMérieux, Bagno a Ripoli (FI) Italy). Intestinal viruses were detected through PCR analysis of stool samples (BIOFIRE, FILMARRAY Gastrointestinal Panel, BioMérieux), and virological serology tests were conducted on blood samples. Finally, our focus narrowed to pediatric patients diagnosed with COVID-19 and myositis, excluding those with other co-infections.

### 2.3. Acquisitions Dataset

Data such as demographics, clinical findings, and laboratory results were extracted from the medical records. Additionally, information on recurrence, complications, treatments, and outcomes was recorded. For the diagnosis and monitoring of myositis, parameters such as muscle weakness, muscle pain (myalgia), elevated levels of muscle enzymes (e.g., creatine kinase or CK), electromyography (EMG) findings, magnetic resonance imaging (MRI) of muscles, and muscle biopsy results were considered. We performed a muscle MRI on only one patient who experienced pain localized exclusively to the lower extremity, which revealed patchy infiltration of the muscle and heterogeneous or diffuse enhancement of the adductor brevis and adductor magnus. The study received approval from the local Ethics Committee, and informed consent was obtained from all the parents of the subjects involved in the study.

#### 2.3.1. Primary Outcome

In evaluating the risk of acute renal injury, we propose that, in patients with mild symptoms, serum creatine kinase (CK) levels elevated to more than 10 times the normal values, and laboratory signs of impaired nitrogen metabolism, should be considered at risk.

#### 2.3.2. Statistical Analyses

Data were presented as medians and ranges for continuous variables. Categorical variables, such as sex (M/F = 7/3), and clinical presentations were represented as absolute numbers. The association between variables was determined using Spearman’s rank correlation coefficient, particularly between CK normalization days and the urea/creatinine ratio (UCR) at admission. 

**Figure 2:**
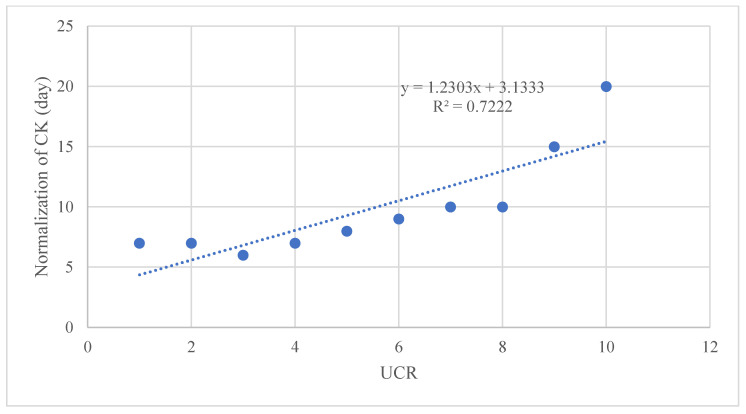
Linear regression graph, showing a correlation between the number of days necessary for the normalization of CK levels and the UCR ratio (R^2^ = 0.7222).

**Figure 3:**
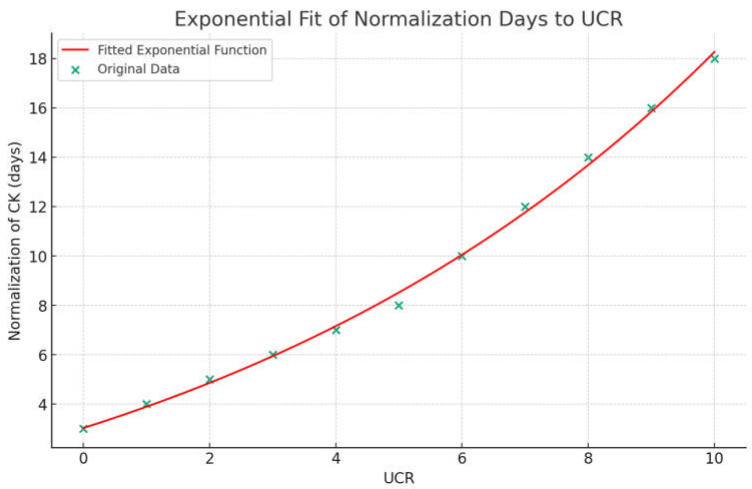
Exponential function curve between UCR values and days for CK normalization. Normalization of CK (days) = 7.08 × e^(0.1148 × UCR)^ − 4.05.

**Figure 4:**
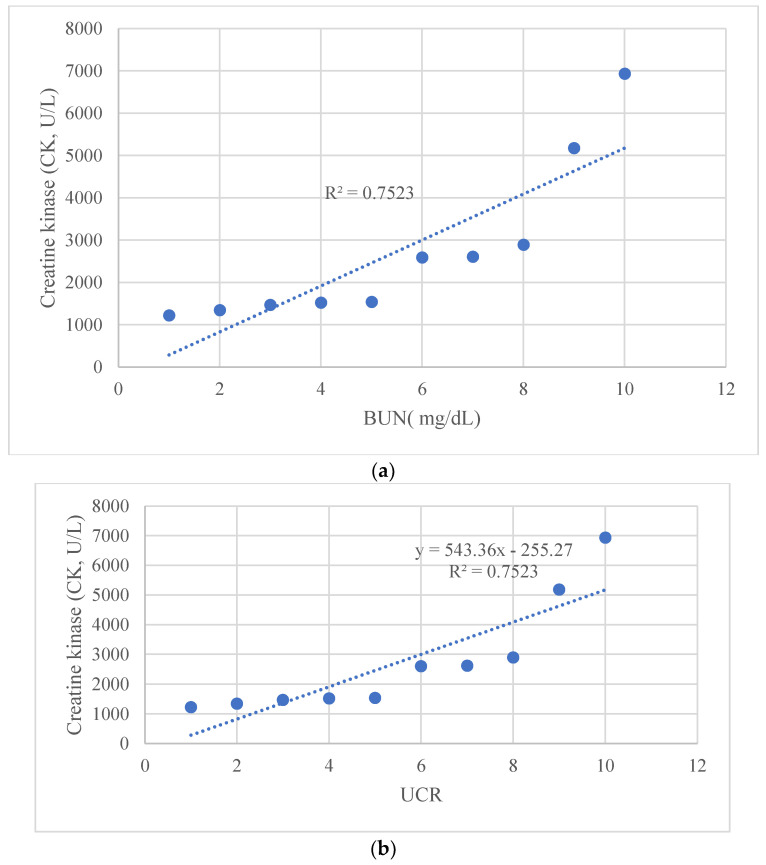
Linear regression graph, showing a correlation (**a**) between CK levels and BUN (mg/L), and (**b**) between CK levels and UCR ratio.

The graphs show (a) the direct linear correlation between CK levels and BUN (mg/L), and (b) between CK levels and UCR ratio. Furthermore, we calculated an R-squared value close to unity in all investigated relationships. The R square measures the strength of the linear relationship between the independent variables included in the regression model and the dependent variable. Stronger relationships, R∼1, indicate less dispersion of the data around the regression line. The significance of the relationship between maximum CK levels with both BUN and UCR was analyzed using linear regression models. The significance level was set at *p* < 0.05. All statistical analyses were performed using the Statistical Package for the Social Sciences (SPSS) version 26. Figure 2, Figure 3 and Figure 4 depict the relationships as mentioned earlier, and significance was denoted with an asterisk (*) where applicable. The descriptive statistics on demographic data, clinical features, laboratory findings, and treatment outcomes were tabulated and summarized to give a comprehensive view of the study’s patient cohort and their clinical progression during hospitalization.

## 3. Results

### 3.1. Demographic Data

From January 2022 to January 2023, ten eligible patients with myositis and SARS-CoV-2 infection were admitted to the pediatric ward or to the Enhanced Centralized Quarantine Center operated by Policlinico “G. Rodolico-San Marco” University Hospital in Catania. The cohort consisted of seven males and three females, with a median age of 7.5 years (range 6–12 years) (Table 1). Most of these cases (70%) occurred during the winter, with the remaining 30% in the summer. No patient was vaccinated for SARS-CoV-2. All patients had fever upon SARS-CoV-2 diagnosis and had an uneventful medical history prior to admission. Eight patients did not display muscle weakness and had normal knee-jerk reflexes. However, two patients showed reduced muscle strength. 

### 3.2. Clinical Features

All patients reported febrile prodromes, symmetrical calf muscle pain, and an abnormal gait. At the initial examination, 30% declined to bear weight on their legs, with many described as having general motor impairment.

### 3.3. Laboratory Findings

At presentation, the median serum CK was 2733 U/L (normal values < 150 U/L, range 1225–6937 U/L). The median absolute leukocyte count stood at 6176 elements/μL, with 20% of patients showing neutropenia (neutrophils < 1500/μL). Hemoglobin and platelet counts were within the standard range for all patients. Renal function, indicated by blood urea nitrogen and creatinine, was monitored over time (Table 1). There was no elevation in CK-MB or troponin-I. Urine tests were all negative for bacteria, leukocytes, and nitrates; hence, urine microscopy was not conducted. The nasopharyngeal samples from all patients tested positive solely for SARS-CoV-2 via multiplex PCR.

### 3.4. Treatment and Outcome

Patients received intravenous normal saline hydration (with a 1.2–1.5 ratio compared to daily maintenance fluid) for 2–3 days and paracetamol for pain relief as required. The CK levels peaked between days 5 and 7 after the onset of myositis symptoms. All were discharged within a week without complications.

Using cut-offs described by Bohn et al. [14,24] (Table 2), we observed correlations between CK normalization days and the urea/creatinine ratio (UCR) at admission (Figure 2 and Figure 3). Additionally, a significant relationship was found between maximum CK levels with both BUN and UCR (Figure 4). 

All patients were hospitalized and treated as required. There were no incidents of myoglobinuria or renal/hydroelectrolytic abnormalities during their stay. On average, hospitalization lasted 7 days. Post-discharge, patients were referred to family pediatricians for clinical evaluations and serum CK level follow-ups. Home recommendations included oral analgesics, rest, and adequate fluid intake. All children experienced clinical and laboratory improvement with no residual complications. No returns to the emergency department were reported in the subsequent month.

## 4. Discussion

We report, for the first time, a correlation between the number of days necessary for the normalization of CK levels and the UCR ratio (R^2^ = 0.7222), CK levels and BUN (mg/L), and between CK levels and the UCR ratio. The predictive value of the UCR ratio and the other statistical associations need to be further investigated to strategically prevent uneventful clinical events, such as myoglobinuria and serious renal involvement. Furthermore, we might speculate that if the UCR, BUN, or UCR values are above a certain threshold, the recovery time could be prolonged.

In our study, after treatment and appropriate fluid management, along with very close monitoring, the children showed progressive clinical and laboratory improvement with complete recovery and no recurrence.

In our ward, none of the pediatric patients with SARS-CoV-2 infection and myositis developed myolysis. We observed no metabolic alterations typically associated with myolysis in any of the patients examined. Specifically, there were no significant changes in aldolase and lactate dehydrogenase levels, nor in electrolytes that would suggest myolysis. We also did not notice any reduction in muscle mass, and the altered enzymatic values eventually regressed. Clinical and laboratory follow-ups revealed no ongoing reduction in muscle strength. Typically, disruption of skeletal muscle integrity leads to the release of intracellular muscle components into the bloodstream and extracellular space, including myoglobin, creatine kinase (CK), aldolase, and lactate dehydrogenase, along with electrolytes.

Patients treated with sufficient intravenous hydration showed a swift recovery, and subsequent evaluations did not reveal any issues with kidney function. Our findings indicate that myositis tends to be a mild condition with a tendency to resolve without intervention.

In light of the ongoing pandemic, it is crucial to conduct more immunological and histological research to unravel the intricate pathways of muscle degradation triggered by SARS-CoV-2, along with other complex clinical signs.

The association between viral infections and myositis has already been well documented in medical literature. Viruses such as influenza, enterovirus, and adenovirus have been associated with myositis in pediatric populations [25,26,27].

Pediatric myolysis is predominantly linked to viral infections, with cases of myositis related to the influenza virus accounting for the majority (more than 80%) [28].

Literature reported COVID-19-induced myositis as a rare manifestation of COVID-19. It is associated with viral myositis attributable to direct myocyte invasion or the induction of autoimmunity. Virus-mediated muscle inflammation has been attributed to angiotensin-converting enzyme (ACE2) receptor-mediated direct entry and the affliction of muscle fibers, leading to innate and adaptive immune activation [29,30,31].

Infectious myositis is usually a self-limiting occurrence, which is characterized by symmetric lower extremity pain typically affecting school-aged children [32]. The evolution of myolysis into kidney damage is rarely reported. Despite this, the acute presentation commonly concerns both parents and healthcare providers, often conducting unnecessary workups [32].

Nevertheless, the probability of concurrent influenza was deemed low due to minimal influenza cases in the community, which was a result of stringent infection control measures during the pandemic [32,33].

The clinical features observed in patients with SARS-CoV-2 and myositis, such as the age group (predominantly 5–9 years old), male preponderance, muscle pain primarily in the lower limbs during recovery, and the non-severe, self-limiting nature of the condition, align with those seen in influenza-related myositis or Benign Acute Childhood Myositis (BACM) [32,33,34,35].

Notably, there was no observed decrease in voluntary movement. As well as past BACM observations, none of our patients exhibited myoglobinuria, a marker for potential acute kidney injury [34]. Consequently, for children with BACM, routine checks such as serum creatine kinase (CK) levels, kidney function, and urinalysis, alongside conservative treatment involving pain relief and rest, are generally adequate [35,36]. We believe that myolysis linked to the Omicron variant of SARS-CoV-2 follows a clinical trajectory like that of BACM, where, after excluding kidney complications, managing patients on an outpatient basis with regular follow-up proves to be satisfactory. We performed daily monitoring of CK values and various laboratory parameters. We observed that peak CK values correlated with BUN values and with UCR values.

BUN, a waste product of protein metabolism produced in the liver and excreted by the kidney, is often used in combination with creatinine to evaluate renal function in clinical settings. BUN is the main end-product of protein metabolism in the human body and is excreted mainly by the kidneys. The BUN level increases when there is excessive protein breakdown or when the glomerular filtration rate decreases. Thus, the BUN level can reflect protein catabolism in the human body [36], which is also a marker of renal impairment. The rate of protein catabolism increases significantly in patients with sepsis [36], and sepsis is often complicated by acute renal injury [37].

UCR, also known as the BUN/creatinine ratio, is a calculated laboratory value that is used to assist in the differential diagnosis of kidney dysfunction and other medical conditions. It is particularly important in predicting prerenal injury, commonly referred to as prerenal azotemia, which is characterized by elevated levels of antidiuretic hormone, increased urea reabsorption, and a reduced glomerular filtration rate.

Pearson’s correlation test showed a close correlation between the maximum CK level during myositis and the maximum level of BUN and UCR. In three out of ten patients, we found that these values were above the normal range according to the cut-offs for BUN and UCR described by Bohn et al. [24] for pediatric patients. We used their values to interpret the renal function data. We observed that patients who had higher BUN and UCR values had a longer time to normalize CK values. We then performed a broad search in the literature to determine if these correlations had been described. To our knowledge, this is the first time that the correlation between increased BUN and BUN/creatinine values to the trend of CK values during an episode of COVID-19 myositis is described.

The strengths of this study lie in its innovative approach to understanding the interactions of SARS-CoV-2 with other health parameters. We utilized cutting-edge technologies and methodologies that allowed for an in-depth analysis not seen in many contemporaneous studies. Furthermore, the integration of multi-disciplinary perspectives enriched the interpretation of the findings.

However, while offering these valuable insights, the study has several limitations. Firstly, our sample size was limited, which might affect the generalizability of the findings. In addition, due to the small cohort, the data we generated may give the impression of cut-off values or an exponential relationship; however, this needs further confirmation with a larger sample size. Moreover, given the currently available data and our research efforts, differentiating between myositis related to COVID-19 and that caused by other viruses remains a significant challenge. This difficulty arises from the overlapping clinical features and pathophysiological mechanisms. Further studies should focus on this aspect, aiding in the differentiation of these forms. Furthermore, elucidating or demonstrating the link between SARS-CoV-2 infection and the occurrence of myositis is beyond the scope of our study. Our focus was primarily on the treatment and outcomes of viral myositis in patients with SARS-CoV-2 infection.

The retrospective nature of the study may introduce biases as we relied on previously recorded data. Moreover, potential co-infections were not exhaustively examined for all participants, which could be pivotal in understanding the full clinical picture.

In the future, incorporating a larger, multicenter cohort could provide more robust evidence. Prospective designs, with standardized diagnostic and monitoring protocols, would offer more control over variables and reduce potential biases. Lastly, comprehensive screening for other co-infections in patients could offer a clearer understanding of the interactions between SARS-CoV-2 and other pathogens.

## 5. Conclusions

In this study, we delved into the issues of myolysis in children afflicted with myositis and SARS-CoV-2 infection, discerning the diagnostic potential of certain biochemical markers. To underscore, our findings suggest that monitoring CK, BUN, and the BUN/creatinine ratio in children with myositis is pivotal, not merely as indicators of organ damage but also as valuable prognostic markers. Evidently, in our patient series, elevations in these parameters were tied to a more protracted regression of CK, signaling a slower recovery trajectory.

Given the physiological roles of these biomarkers, their relevance in the context of myositis becomes even more pronounced. BUN and its ratio with creatinine, in particular, are instrumental in gauging renal function and potential disruptions to protein metabolism. Since renal complications can aggravate the clinical course of myositis, close vigilance of these renal markers becomes imperative. This monitoring could potentially aid in early interventions and prevent long-term sequels.

In the absence of a proven method of predicting the risk of acute renal failure, we propose that patients with mild symptoms, a normal urine dipstick, and serum CK levels more than 10× normal associated with increased BUN and BUN/creatinine ratio values have to be considered low-risk patients.

Moreover, our observations also draw attention to the nuances of SARS-CoV-2-exposed myositis in children. Its clinical presentation, bearing resemblance to influenza-associated myositis or BACM, hints at underlying similarities in pathophysiology. The uniqueness of our findings, being the first to correlate the dynamic of BUN and BUN/creatinine with the trend of CK in the backdrop of COVID-19 myositis, offers a fresh perspective and a foundation for future inquiries.

However, while these insights paint a clearer picture of the interplay between SARS-CoV-2 and myolysis, a more panoramic view necessitates a larger sample size. This would not only provide more definitive results but also allow for a more comprehensive understanding of the varied clinical presentations. By juxtaposing SARS-CoV-2-positive myositis in children with myositis resulting from other viral etiologies, the peculiarities and commonalities can be illuminated, paving the way for tailored therapeutic interventions.

Additionally, the continuous evolution of the COVID-19 pandemic and the emergence of new variants raises a pertinent question: does the clinical picture and association between these biomarkers change with different strains of the virus? Further research is warranted to elucidate this.

Furthermore, we authors would like to underline the need for double-blind studies to better understand the similarities and differences between COVID and non-COVID populations.

## Figures and Tables

**Table 1:** Characteristics of the population and laboratory parameters.

Patient Number	1	2	3	4	5	6	7	8	9	10
Age (years)	7	5	6	12	8	6	8	9	7	5
Sex	M	M	M	F	M	M	M	M	F	F
White blood cell count (cells /µL)	7220	5450	5710	7220	6170	4220	6940	4567	5620	8650
Hemoglobin (g/dL)	13	12.5	11.6	13	13.7	112	14	13.5	13.9	12.3
Platelet count (×10^3^/µL)	350	178	277	250	250	227	121	231	201	231
Creatine kinase (CK, U/L)	1225	1346	1475	1524	1543	2596	2614	2892	5180	6937
Blood urea nitrogen (BUN, mg/dL)	6	8.64	9.74	10.85	13.8	16.28	19	22.29	26	35.75
Serum creatinine (mg/dL)	0.26	0.32	0.34	0.35	0.46	0.46	0.38	0.49	0.45	0.55
UCR	23	27	28.7	31	30.5	35.4	50	45.5	58.7	65
Normalization of CK (day)	7	7	6	7	8	9	10	10	15	20
Aspartate aminotransferase (AST, U/L)	46	112	58	53	76	35	55	65	89	150
Alanine aminotransferase (ALT, U/L)	35	25	19	20	23	15	16	20	29	34
C-reactive protein (CRP, mg/dL)	2.41	2.36	4.77	4.53	5.29	3.19	5.83	9.74	7.11	6.59
Serum sodium (mmol/L)	138	137	139	140	141	145	137	144	142	143
Serum potassium (mmol/L)	4.5	4.2	3.8	3.7	4.1	3.9	3.8	3.9	4.4	4.2

**Table 2:** Pediatric reference intervals for the urea creatinine ratio from Bohn, M.K et al. [24].

			Urea Creatinine Ratio (SI Units)		BUN Creatinine Ratio (CON Units)	
Assay	Age Partition	n	Lower Reference Limit	Upper Reference Limit	Lower Reference Limit	Upper Reference Limit
Creatinine (enzymatic)	0 to <15 days	51	21	162	5	40
	15 days to <1 year	126	49	438	12	108
	1 to <3 years	59	127	419	31	1
	3 to <5 years	89	130	299	32	74
	5 to <8 years	115	87	246	22	61
	8 to <10 years Female	57	69	177	17	44
	8 to <10 years Male	41	83	189	21	47
	10 to <15 years	272	50	146	12	36
	15 to <19 years	228	44	107	11	26
Creatinine (Jaffe)	0 to <15 days	74	26	121	6	30
	15 days to <1 year	120	35	156	9	39
	1 to <5 years	148	70	198	17	49
	5 to <8 years	113	69	160	17	40
	8 to <10 years Female	57	57	131	14	32
	8 to <10 years Male	42	66	156	16	39
	10 to <15 years Female	147	42	111	10	27
	10 to <15 years Male	130	50	134	12	33
	15 to <19 years	229	42	99	10	25

## Data Availability

The data presented in this study are available on request from the corresponding author.
